# Determination of Anti-nuclear Antibody Pattern Distribution and Clinical Relationship

**DOI:** 10.12669/pjms.302.4276

**Published:** 2014

**Authors:** Zafer Mengeloglu, Tekin Tas, Esra Kocoglu, Gülali Aktas, Seyda Karabörk

**Affiliations:** 1 Zafer Mengeloglu, MD, Assistant Professor, Department of Medical Microbiology, Abant Izzet Baysal University School of Medicine, Bolu, Turkey.; 2 Tekin Tas, MD, Assistant Professor, Department of Medical Microbiology, Abant Izzet Baysal University School of Medicine, Bolu, Turkey.; 3 Esra Kocoglu, MD, Associate Professor, Department of Medical Microbiology, Abant Izzet Baysal University School of Medicine, Bolu, Turkey.; 4 Gülali Aktas, MD, Assistant Professor, Department of Internal Diseases, Abant Izzet Baysal University School of Medicine, Bolu, Turkey.; 5 Seyda Karabörk, Specialist of Medical Microbiology, Department of Medical Microbiology, Abant Izzet Baysal University School of Medicine, Bolu, Turkey.

**Keywords:** Anti-nuclear antibody, ANA, IIF, Immunfluorescence

## Abstract

***Background and Objectives:*** Autoantibodies are immunglobulins occurred directly against autoantigens that are known as endogen antigens. Autoimmune disease is an occasion that the body begins a fight against its own cells and tissues. The antibodies that are created by the body against its own cell nuclei are called as anti-nuclear antibodies (ANA), and one of the methods used for detection and pattern of ANA is indirect immunofluorescence test (IIF). In the present study, it was aimed to determine the rate of ANA positivity and patterns of the positive specimens, and to investigate the relationship between ANA positivity and diseases in patients.

***Methods:*** ANA test results of a total of 3127 patients admitted during March 2010 to December 2012 were evaluated retrospectively. ANA test (HEp 20-10, EUROIMMUN, Germany) was used in dilution of 1:100 in IIF test.

***Results:*** A total of 494 (15.8%) resulted as ANA positive. ANA positivity rate was significantly higher in female patients than the male ones (p<0.001). The most frequent ANA patterns were coarse speckled pattern (154 patients, 31.2%), nucleolar pattern (89 patients, 18.0%), fine speckled pattern (57 patients, 11.5%), and speckled pattern (48 patients, 9.7%). ANA positivity was most commonly determined in rheumatoid arthritis (RA) (42 patients, 8.5%), systemic lupus erythematosus (SLE) (29 patients, 5.9%), and rheumatoid vasculitis (RV) (28 patients, 5.7%). The most frequent symptoms or findings were joint pain (127 patients, 26.0%) and anemia (28 patients, 5.7%). ANA positivity rates were found to be significantly higher in patients with RA (p<0.001), with SLE (p<0.001), and with Raynaud phenomenon (p=0.001) in comparison to the controls. Amongst the most frequent diseases evaluated, no significant differences were found between the control groups and the groups of RV (p=0.089), multiple sclerosis (p=0.374), and Sjögren syndrome (p=0.311) in terms of ANA positivity rates.

***Conclusions:*** The present study is the first study reporting the positivity rate and distribution of ANA in Bolu located in northwestern Turkey. Information about the pattern types and the distribution of the patterns according to the diseases and symptoms contribute in diagnosis of autoimmune diseases. It is observed that clinical diagnosis has been supported significantly by ANA test according to data of our study.

## INTRODUCTION

Autoantibodies are immunglobulins occurred directly against auto antigens that are known as endogen antigens. Autoantibodies can occur against proteins such as intracellular enzymes, receptors, structural elements, glycoproteins, phospholipids, and nucleic acids. Autoimmune disease is a disorder in which the body begins a fight against its own cells and tissues. It was reported that autoimmune disorders effected about 3% of North America and Europe, and that more than 75% of those were females.^1^^,^^2^

The antibodies that are created by the body against its own cell nuclei are called as anti-nuclear antibodies (ANA). It is not clear how the body start a fight against its own cells, however, several studies have reported that this was associated with some factors such as genetic characteristics, infections and/or environmental factors. Autoimmune diseases have various symptoms and findings. These may be classified as organ-specific and systemic autoimmune disorders. Some of these are insulin-dependent diabetes mellitus, rheumatoid arthritis (RA), systemic lupus erythematosus (SLE), scleroderma and multiple sclerosis (MS). The most common targets of autoimmune diseases are thyroid gland, stomach, adrenal glands, and pancreas. Systemic autoimmune diseases effect skin, joints and muscle tissues. Autoantibodies interfere in this situation.^1^^-^^3^ Autoantibody detection in serum samples plays important role in diagnosis and in follow-up of autoimmune diseases for the last 50 years. Commercial kits have been developed for this purpose. Amongst these kits, IIF is used for screening test. IIF test determines the presence of anti-nuclear antibodies (ANA).^3^^,^^4^ In cases that ANA test result is insufficient for the diagnosis, disease-specific antigen screening is suggested to be performed. Besides this, detection of ANA positivity in healthy individuals in the community shows the need for studies conducted on the prevalence.^3^^-^^5^

In the present study, it was aimed to determine the rate of ANA positivity and patterns of the positive specimens, and to investigate the relationship between ANA positivity and diseases in patients whose sera were tested for ANA using IIF in the microbiology laboratory of our hospital.

## METHODS

***Patients and ANA tests: ***In the study, ANA test results of a total of 3127 patients admitted during March 2010 to December 2012 were evaluated retrospectively. ANA test (HEp 20-10, EUROIMMUN, Germany) was used in dilution of 1:100 in IIF test. The slides prepared following the recommendations of the manufacturer were evaluated under the fluorescence microscope using 40X objectives. Positive and negative controls were used in every evaluation. Fluorescence intensity was interpreted semi quantitatively based on negative control (0) and positive control (+4). This study was approved by the local ethics committee.

***Statistical analysis: ***A statistical analysis was performed using IBM SPSS Statistics Version 15.0 (SPSS Inc., Chicago, IL, United States). Descriptive variables were presented as numbers and percentages. For the categorical variables, differences between groups and associations between the variables were analysed using Chi Square test. An independent sample T test was used for comparison between the groups for the comparison in terms of age. The results were evaluated within a confidence interval of 95%, and a p value of less than 0.05 was considered statistically significant. 

## RESULTS

Amongst 3127 tests, 494 (15.8%) resulted as ANA positive. The mean age was 43.2±18.0 in ANA-positive patients and was 42.5±17.0 in ANA-negative individuals, and the difference between the groups was not significant (p=0.408).

Amongst the patients, 1141 (33.6%) were males and 2076 (66.4%) were females. ANA was positive in 125 (11.0%) of the males and 369 (17.8) of the females; according to this finding ANA positivity rate was significantly higher in female patients than the male ones (p<0.001).

The most frequent ANA patterns were coarse speckled pattern (154 patients, 31.2%), nucleolar pattern (89 patients, 18.0%), fine speckled pattern (57 patients, 11.5%), and speckled (granular) pattern (48 patients, 9.7%). ANA positivity was most commonly determined in RA (42 patients, 8.5%), SLE (29 patients, 5.9%), and rheumatoid vasculitis (RV) (28 patients, 5.7%).The most frequent symptoms or findings were joint pain (127 patients, 26.0%), anemia (28 patients, (5.7%), transaminase elevation (13 patients, 2.6%), and neurological symptoms (12 patients, 2.4%) (Table-I). The distribution of ANA patterns in terms of diseases and findings is shown on Table-I.

ANA positivity was observed in 34.9% (29/83) of SLE patients, 26.3% (42/160) of RA patients, 32.1% (17/53) of Raynaud patients, and 22.3% (7/31) of Sjögren syndrome patients (Table-II).

ANA positivity rates were found to be significantly higher in patients with RA (p<0.001), with SLE (p<0.001), and with Raynaud phenomenon (p=0.001) in comparison to the controls. No significant differences were found between the control groups and the groups of RV (p=0.089), multiple sclerosis (p=0.374), and Sjögren syndrome (p=0.311) in terms of ANA positivity rates (Table-II).

## DISCUSSION

IIF plays an important role in diagnosis of autoimmune diseases. Determination of ANA patterns using IIF is useful in differential diagnosis. ANA tests are easy to perform and have low cost, however, they have negative aspects such as subjectivity and need for experienced staff in the interpretation, and low sensitivity and specificity. Despite this, ANA tests have remained important because particularly some of the patterns are extremely helpful in the diagnosis and in some cases the clinician needs to be supported by the IIF result.^5^^-^^8^

**Table-I:** Distribution of anti-nuclear antibody patterns according to some of common diseases and findings

*Anti-nuclear antibody patterns*	*Total*	*Diseases (n)*								*Symptoms and findings (n)*					
		*RA*	*SLE*	*Rheumatoidvasculitis*	*Raynaudphenomenon*	*MS*	*Sjögrensyndrome*	*Unknown andothers*		*Joint pain*	*Anemia*	*Transaminase disorder*	*Neurologicalsymptoms*	*Alopesi*	*Unknown andothers*
Coarse speckled pattern	154	18	6	8	6	5	2	109		40	15	2	4	4	89
Nucleolar pattern	89	1	4	4	2	1		77		27	4	3	2	2	51
Fine speckled pattern	57	6	2	1	2		2	44		13	3	4	2	1	34
Speckled (Granular) pattern	48	3	6	7	2	2		28		7	1	2	1	1	36
Nuclear dots	31	4	1		1		1	24		10	3	1	1		16
Centromere pattern	23	5		1	2		2	13		5	1				17
Homogeneous pattern	20	1	5	1				13		7	1	1			11
Peripheral pattern	14	1	1	2	1			9		2			1		11
Speckled and speckled chromosomal staining	8					1		7		3					5
Homogeneous and speckled pattern	7	3	1	1				2							7
Speckled pattern and chromosomal staining	6							6		2					4
Other	37	0	3	3	1	1	0	29		11	0	0	1	0	25
Total	494	42	29	28	17	10	7	361		127	28	13	12	8	306

RA: Rheumatoid arthritis; SLE: Systemic lupus erythematosus; MS: Multiple sclerosis.

**Table-II:** Comparisons between anti-nuclear antibody positivity and some of common diseases evaluated in the study

*Disease*	*Result*	*Anti-nuclear antibody*					*Total (n=3217)*	*P*
		*Positive (n=494)*			*Negative(n=2723)*			
		*n*	*%*		*n*	*%*		
RA	Positive	42	26.3		118	73.7	160	<0.001
	Negative	452	14.8		2605	85.2	3057	
								
SLE	Positive	29	34.9		54	65.1	83	<0.001
	Negative	465	14.8		2669	85.2	3134	
								
RV	Positive	28	11.6		214	88.4	242	0.089
	Negative	466	15.7		2509	84.3	2975	
								
RP	Positive	17	32.1		36	67.9	53	0.001
	Negative	477	15.1		2687	84.9	3164	
								
MS	Positive	10	11.9		74	88.1	84	0.374
	Negative	484	15.4		2649	84.6	3133	
								
Sjögren S.	Positive	7	22.6		24	77.4	31	0.311
	Negative	487	15.3		2699	84.7	3186	

RA: Rheumatoid arthritis; SLE: Systemic lupus erythematosus;

RV: Rheumatoidvasculitis; RP: Raynaud phenomenon; MS: Multiple sclerosis.

In the present study, ANA positivity rate, distribution of ANA patterns according to the symptoms and findings, and the association of the patterns with some diseases were evaluated. The most frequently reported patterns were speckled (Granular) and nucleolar patterns in concordant with some other studies.^9^^,^^10^ Speckled patterns were the most common patterns. Yilmaz et al.^11^ reported homogenous pattern in more than half of their patients.

In this study, significant associations were found between ANA positivity and RA, SLE and Raynaud phenomenon. However, no significant relationships were detected between ANA positivity and RV, multiple sclerosis and Sjögren Syndrome. In the analysis, some diseases such as diabetes mellitus, hypertension and heart failure and some findings such as anemia and joint pain were excluded in order to avoid false results. Because these diseases are not found solely in a patient, and these findings are not unique for a specific disease.^3^^-^^5^ In addition, we couldn’t reach clinical symptoms and findings of some patients included in the study due to the nonspecific record of the data.

ANA positivity was reported to be found in 5% of healthy individuals due to unknown reasons. It was reported that this rate could reach to 15% in further ages.^12^^-^^14^ Tan et al.^12^ detected ANA positivity rate as 31.7% in healthy individuals using a dilution of 1:40 of the sera. It is suggested that test should be repeated in a patient that were positive for ANA after a 3-6 months period when the symptoms of the disease disappear. A positive result in this repeat test would be supportive for the clinical diagnosis, however a negative result could mean that the first positivity might be due to polyclonal B activation.^5^^,^^12^ Positive results that were provided by our study and that did not seem to be directly related to the disease can be explained in this way.

It was noted that ANA test was positive in connective tissue disorders, however, it was added that ANA positivity was not absolutely diagnostic. It was reported that ANA positivity could be observed in 95% in SLE and mixed connective tissue disorders, and that this rate could be seen between 25-70% of the cases with Sjögren syndrome, systemic sclerosis and RA.^1^^,^^4^ In the present study, ANA positivity was found in a low rate as in 34.9% of SLE patients. In addition, it was observed in a rate between 22-26% in patients with Sjögren Syndrome and RA.

In conclusion, this is the first study reporting the positivity rate and distribution of ANA in Bolu located in northwestern Turkey. Information about the pattern types and the distribution of the patterns according to the diseases and symptoms contribute in diagnosis of autoimmune diseases. It is observed that clinical diagnosis has been supported significantly by ANA test according to data of our study.

## Authors Contribution:

**ZM:** Substantial contributions to conception and design, or acquisition of data, or analysis and interpretation of data; final approval of the version to be published.

**TT:** Drafting the article or revising it critically for important intellectual content.

**EK:** Substantial contribution to data collection, analysis and interpretation, drafting of article.

**GA:** Acquisition of data and drafting the article.

**SK:** Substantial contribution to data collection and drafting the article.

## Notes

***Source of funding:*** None to declare.
